# Work-related physical activity among adults in Germany

**DOI:** 10.17886/RKI-GBE-2017-039

**Published:** 2017-06-14

**Authors:** Jonas D. Finger, Gert B. M. Mensink, Cornelia Lange, Kristin Manz

**Affiliations:** Robert Koch Institute, Department for Epidemiology and Health Monitoring, Berlin, Germany

**Keywords:** PHYSICAL INACTIVITY, WORK, SITTING, ADULTS, HEALTH MONITORING

## Abstract

In GEDA 2014/2015-EHIS the prevalence of work-related physical activity was estimated based on respondents’ self-reported data. 47.5% of women and 47.2% of men mostly sit or stand during work. The highest proportion of people who mostly sit or stand during work is found among 18- to 29-year-old women (55.5%) and men aged 30 to 44 (50.2%). A significantly higher proportion of men (14.8%) than women (3.2%) have jobs involving mostly heavy manual labour. For both genders, the higher a person’s level of education, the more likely it is that physical activity during work is limited to sitting or standing. The results highlight great potential to promote physical activity.

## Introduction

Physical activity is any movement by the skeletal muscles that increases the body’s energy expenditure beyond the basal metabolic rate (BMR) [1]. Physically non-demanding activities performed whilst sitting or standing hardly raise energy expenditure beyond the BMR [2]. Sitting for long hours, as is normal in office jobs, constitutes a risk factor for non-communicable diseases [3, 4]. According to current estimates, the general mortality risk for adults increases by 2% for every hour spent sitting per day [4]. Where employment involves physical activity, such as for example in agriculture, work-related physical activity is often a person’s greatest expenditure of energy, as working days usually comprise eight-hour shifts [5]. Whilst work-related physical activity has health benefits, these are not as great as the health benefits of aerobic physical exercise during leisure time [6-8]. The reason is that work-related physical activity is often repetitive, and usually involves working overhead and carrying heavy objects. This can increase muscular strength, yet hardly improves aerobic endurance capacity [9-11]. Endurance capacity improves during aerobic leisure activities such as jogging and swimming, and is particularly important with regard to preventing non-communicable diseases (such as cardiovascular diseases, certain types of cancer or diabetes) and their underlying cardiometabolic risk factors (such as hypertension, lipometabolic disorders and obesity) [12]. Due to the high relevance of physical inactivity as a contributing factor to disease development, the World Health Organization (WHO), in its Global Action Plan for the Prevention and Control of Non-Communicable-Diseases 2013-2020, established the goal of a 10% relative reduction in prevalence of insufficient physical activity by 2025 (compared with 2010) [13].

**GEDA 2014/2015-EHIS**

**Data holder:** Robert Koch Institute

**Aims:** To provide reliable informa tion about the population’s health status, health-related behaviour and health care in Germany, with the possibility of a European comparison

**Method:** Questionnaires completed on paper or online

**Population:** People aged 18 years and above with permanent residency in Germany

**Sampling:** Registry office sample; randomly selected individuals from 301 communities in Germany were invited to participate

**Participants:** 24,016 people (13,144 women; 10,872 men)

**Response rate:** 26.9%

**Study period:** November 2014 - July 2015

**Data protection:** This study was undertaken in strict accordance with the data protection regulations set out in the German Federal Data Protection Act and was approved by the German Federal Commissioner for Data Protection and Freedom of Information. Participation in the study was voluntary. The participants were fully informed about the study’s aims and content, and about data protection. All participants provided written informed consent.

More information in German is available at www.geda-studie.de

## Indicator

Using a validated German version of the European Health Interview Survey – Physical Activity Questionnaires (EHIS-PAQ), the German Health Update (GEDA 2014/2015-EHIS) survey for the first time measured work-related physical activity [14, 15]. In GEDA 2014/2015-EHIS, respondents were asked: ‘When you work, what best describes what you do? (a) mostly sitting or standing; (b) mostly walking or tasks of moderate physical effort; (c) mostly heavy labour or physically demanding work, or (d) not performing any working tasks.’ Work, here, encompasses not only paid but also unpaid work (for example, studying or housework). Respondents were asked to select only one answer. For the purpose of the analysis presented here, these four answers on work-related physical activity for the 18-to-64 age group were stratified by gender, age group, level of education and federal state. A statistically significant difference between groups is assumed when confidence intervals do not overlap.

The analyses are based on the data received from 18,026 participants of working age, aged 18-to-64 (10,146 women and 7,880 men) with valid data in EHIS-PAQ. Calculations were carried out using a weighting factor that corrects for deviations within the sample from the German population (as of 31 December 2014) with regard to gender, age, community type and education. The community type accounts for the degree of urbanisation and reflects the regional distribution in Germany.

The International Standard Classification for Education (ISCED) was used to ensure that the responses provided on educational levels were comparable [16]. A detailed description of the methodology applied in the GEDA 2014/2015-EHIS study can be found in the article German Health Update – New data for Germany and Europe in issue 1/2017 of the Journal of Health Monitoring.

## Results and discussion

**Figure 1:**
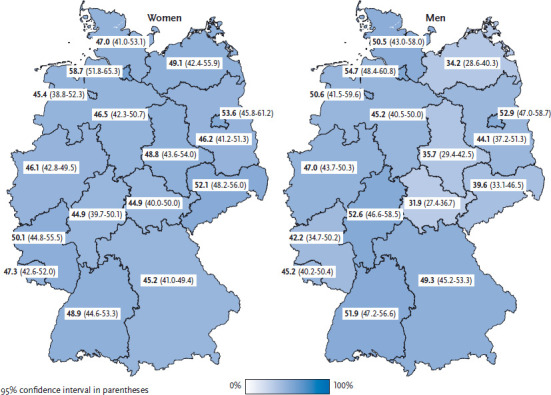
Physical activity during work according to gender and German federal state (n=10,146 women; n=7,880 men) Source: GEDA 2014/2015-EHIS

Nearly half of all women (47.5%) and men (47.2%) of working age (18 to 64) stated that they sit or stand most of the time during work and therefore spend many hours per day physically inactive. Among women, the prevalence of work-related physical inactivity (mostly sitting or standing) is highest in the 18-to-29 age group (55.5%) (Table 1). Among men, it is highest in the 30-to-44 age group (50.2%) (Table 2). Compared with women, men nearly five times as often reported being employed in jobs that involve mostly heavy manual labour. The observed regional and educational differences in work-related physical activity are stronger among men than among women (Table 1 and Table 2; Figure 1). In all age groups, men with higher levels of education responded nearly twice as often as men with lower levels of education that they mostly sit or stand during work. The highest value for mostly sitting or standing during work was found in 30- to 44-year-old men with higher education levels (79.7%). Conversely, men with lower education levels are seven times as likely to state that their work implies heavy manual labour than those with higher levels of education. The same is true for women: the higher their level of education, the more likely they are to work sitting or standing.

For women in Hamburg, the amount of work-related physical inactivity is statistically significantly higher than the German average. For men in Thuringia, Mecklen-burg-West Pomerania, Saxony-Anhalt and Saxony, the prevalence of work-related physical inactivity is statistically significantly lower than the German average (Figure 1).

People with higher levels of education, who are often physically inactive during work, engage more often in physical exercise during their leisure time and thereby partially compensate for their lack of physical activity at work [17-19]. However, only high levels of physical activity during leisure time can actually compensate for the negative effects of mostly sitting at work, and the necessary high levels of leisure time physical activity are often not achieved [20]. Integrating physical activity into work routines, for example during breaks and providing exercise classes, should therefore become an important feature of health promotion at the workplace [21]. Those employed in jobs involving heavy manual labour, however, are usually less active during their leisure time [17]. For such people, health-enhancing aerobic physical activities (endurance activities) during leisure time can nonetheless be beneficial, as such type of exercise improves cardiorespiratory fitness, which is only insufficiently promoted by anaerobic manual labour that mainly improves muscular strength. However, people whose work implies heavy manual labour also need to recover physically during their leisure time. The observed regional differences between federal states in terms of work-related physical activity to a certain degree reflect the regional importance of the services industry. In urban agglomerations, the services sector is larger than in less densely populated regions, and the amount of work conducted mostly sitting or standing is higher than in the industrial or agricultural sector. The regional importance of the services sector in 2013 by federal state [22] is more or less congruent with the share of people whose work-related physical activity is limited to sitting or standing in GEDA 2014/2015-EHIS.

With regard to health promotion and prevention, we need to consider that work-related physical activity is primarily determined by individual job requirements. Health promotion at the workplace therefore needs to contribute towards reducing the negative health impacts of work-related physical inactivity. A multi-component approach is recommended, which should include providing exercise classes, changing the workday routine (for example, to include active breaks) and developing exercise-friendly infrastructure (providing bicycle parking, showers, etc.) [21].

## Key statements

- Roughly 47.5% of women and 47.2% of men mostly sit or stand during work.
- The highest proportion of women who mostly sit or stand during work is the 18-to-29 age group (55.5%) and of men, the 30-to-44 age group (50.2%).
- A significantly higher proportion of men (14.8%) than women (3.2%) have jobs involving mostly heavy manual labour.
- In both genders the same pattern applies that the higher a person’s level of education, the more likely it is that physical activity during work is limited to sitting or standing.

## Figures and Tables

**Table 1:** Physical activity during work among women according to age and educational status (n=10,146) Source: GEDA 2014/2015-EHIS

Women	Mostly sitting or standing (physical inactivity)		Mostly walking or tasks of moderate physical effort		Mostly heavy labour or physically demanding work		Not performing any working tasks	
	%	(95% CI)	%	(95% CI)	%	(95% CI)	%	(95% CI)
**Women total**	**47.5**	**(46.1-49.0)**	**40.6**	**(39.0-42.1)**	**3.2**	**(2.8-3.7)**	**8.7**	**(8.0-9.5)**
**18-29 Years**	55.5	(52.6-58.4)	33.7	(30.9-36.7)	3.9	(2.9-5.0)	6.9	(5.6-8.6)
Low education	43.5	(36.9-50.4)	36.5	(30.0-43.5)	3.3	(1.6-6.6)	16.7	(12.5-22.0)
Medium education	55.9	(52.1-59.5)	34.9	(31.3-38.6)	4.9	(3.6-6.6)	4.4	(3.1-6.1)
High education	73.0	(67.9-77.6)	23.9	(19.6-28.8)	0.8	(0.3-2.1)	2.3	(1.2-4.4)
**30-44 Years**	49.5	(47.0-51.9)	42.2	(39.7-44.8)	2.9	(2.3-3.8)	5.4	(4.3-6.8)
Low education	29.7	(23.7-36.6)	50.1	(42.8-57.5)	6.3	(3.5-11.0)	13.8	(9.2-20.3)
Medium education	46.3	(43.2-49.3)	45.7	(42.5-48.8)	3.1	(2.3-4.1)	5.0	(3.9-6.5)
High education	67.8	(63.9-71.4)	29.7	(26.2-33.4)	0.7	(0.4-1.4)	1.8	(1.1-3.0)
**45-64 Years**	42.7	(40.8-44.6)	42.7	(40.8-44.6)	3.1	(2.6-3.7)	11.5	(10.5-12.7)
Low education	24.2	(20.6-28.1)	52.9	(48.5-57.1)	3.8	(2.5-5.9)	19.1	(15.8-22.9)
Medium education	41.7	(39.3-44.0)	43.8	(41.5-46.1)	3.4	(2.7-4.3)	11.2	(9.8-12.7)
High education	62.5	(59.5-65.3)	29.9	(27.4-32.7)	1.4	(0.9-2.1)	6.2	(4.8-8.0)
**Total (women and men)**	**47.3**	**(46.1-48.6)**	**35.6**	**(34.5-36.7)**	**9.0**	**(8.3-9.8)**	**8.0**	**(7.5-8.6)**

**Table 2:** Physical activity during work among men according to age and educational status (n=7,880) Source: GEDA 2014/2015-EHIS

Men	Mostly sitting or standing (physical inactivity)		Mostly walking or tasks of moderate physical effort		Mostly heavy labour or physically demanding work		Not performing any working tasks	
	%	(95% CI)	%	(95% CI)	%	(95% CI)	%	(95% CI)
**Men total**	**47.2**	**(45.6-48.8)**	**30.7**	**(29.3-32.0)**	**14.8**	**(13.5-16.1)**	**7.4**	**(6.6-8.3)**
**18-29 Years**	46.4	(43.5-49.3)	30.4	(27.5-33.5)	16.7	(14.3-19.5)	6.4	(5.0-8.2)
Low education	35.4	(29.1-42.3)	31.6	(25.9-37.9)	18.0	(13.4-23.6)	15.0	(10.7-20.7)
Medium education	44.2	(40.6-47.9)	33.4	(29.5-37.5)	18.9	(15.5-22.7)	3.5	(2.6-4.9)
High education	77.1	(71.2-82.1)	14.8	(11.0-19.8)	5.1	(3.2-8.1)	2.9	(1.0-8.0)
**30-44 Years**	50.2	(47.6-52.9)	30.0	(27.7-32.4)	15.9	(13.9-18.0)	3.9	(2.8-5.4)
Low education	28.2	(21.4-36.1)	37.5	(29.9-45.9)	24.1	(17.7-31.9)	10.2	(6.3-16.2)
Medium education	39.3	(36.0-42.6)	36.0	(32.7-39.4)	20.8	(18.0-23.9)	4.0	(2.6-6.0)
High education	79.7	(76.7-82.4)	16.3	(13.9-19.2)	3.2	(2.1-4.7)	0.8	(0.3-1.9)
**45-64 Years**	45.7	(43.7-47.7)	31.2	(29.4-33.0)	13.2	(11.7-14.8)	10.0	(8.9-11.2)
Low education	29.9	(25.0-35.2)	34.2	(29.1-39.6)	19.8	(16.0-24.3)	16.1	(11.8-21.7)
Medium education	35.2	(32.8-37.8)	36.7	(34.0-39.4)	16.7	(14.4-19.2)	11.4	(9.9-13.2)
High education	70.6	(68.1-72.9)	19.9	(17.8-22.2)	4.3	(3.3-5.6)	5.2	(4.1-6.5)
**Total (women and men)**	**47.3**	**(46.1-48.6)**	**35.6**	**(34.5-36.7)**	**9.0**	**(8.3-9.8)**	**8.0**	**(7.5-8.6)**

CI=confidence interval
